# Multi-scale RNA comparison based on RNA triple vector curve representation

**DOI:** 10.1186/1471-2105-13-280

**Published:** 2012-10-30

**Authors:** Ying Li, Ming Duan, Yanchun Liang

**Affiliations:** 1 College of Computer Science and Technology, Symbol Computation and Knowledge Engineering Lab of Ministry of Education, Jilin University, Changchun, China; 2 Key Laboratory of Zoonoses of Ministry of Education, Jilin University, Changchun, China

**Keywords:** RNA mutation, Secondary structure, RNAdistance, RNApdist, Multi-scale RNA comparison, RNA triple vector curve

## Abstract

**Background:**

In recent years, the important functional roles of RNAs in biological processes have been repeatedly demonstrated. Computing the similarity between two RNAs contributes to better understanding the functional relationship between them. But due to the long-range correlations of RNA, many efficient methods of detecting protein similarity do not work well. In order to comprehensively understand the RNA’s function, the better similarity measure among RNAs should be designed to consider their structure features (base pairs). Current methods for RNA comparison could be generally classified into alignment-based and alignment-free.

**Results:**

In this paper, we propose a novel wavelet-based method based on RNA triple vector curve representation, named multi-scale RNA comparison. Firstly, we designed a novel numerical representation of RNA secondary structure termed as RNA triple vectors curve (TV-Curve). Secondly, we constructed a new similarity metric based on the wavelet decomposition of the TV-Curve of RNA. Finally we also applied our algorithm to the classification of non-coding RNA and RNA mutation analysis. Furthermore, we compared the results to the two well-known RNA comparison tools: RNAdistance and RNApdist. The results in this paper show the potentials of our method in RNA classification and RNA mutation analysis.

**Conclusion:**

We provide a better visualization and analysis tool named TV-Curve of RNA, especially for long RNA, which can characterize both sequence and structure features. Additionally, based on TV-Curve representation of RNAs, a multi-scale similarity measure for RNA comparison is proposed, which can capture the local and global difference between the information of sequence and structure of RNAs. Compared with the well-known RNA comparison approaches, the proposed method is validated to be outstanding and effective in terms of non-coding RNA classification and RNA mutation analysis. From the numerical experiments, our proposed method can capture more efficient and subtle relationship of RNAs.

## Background

RNA once is considered as the fundamental information medium in central dogma of molecular biology. A number of studies have indicated that RNAs play a more active role and carry diverse functionalities in nature, including mediating the synthesis of proteins, regulating cellular activities, and exhibiting enzyme-like catalysis and post-transcriptional activities. Furthermore, many recent discoveries have shown that the number and biological significance of functional RNAs has been underestimated. In living cells, RNAs do not remain in a linear form, which folds its secondary structure through base pairs including canonical bonds of A-U and G-C and wobble pair of G-U. For understanding RNA's functionality, the alignment and similarity of RNA should consider not only the primary structure (sequence) but also the secondary structure (base pairs).

Numerous approaches were proposed to measure the similarity between RNA secondary structures, which can be broadly categorized into two classes: alignment based string or tree representation of RNA secondary structure, and comparison based some numerical representation without alignment.

Most studies usually adopt dynamic programming algorithms and tree models. Some are usually based on the alignment of a string representation of the secondary structures such as the dot-bracket representation, in which a score function or a distance function to represent insertion, deletion and substitution of letters in the compared structures [1-4]. Sequences considered in alignment of RNA secondary structures are not only string sequences but also secondary structure. Different weights or different score functions are designed for unpaired nucleotides and paired nucleotides.

Others are almost based on alignment of a tree representation of the RNA secondary structure elements or the base pairing probability matrices [5-9]. Shapiro [5,6] proposed various tree models used for representing RNA secondary structures without pseudoknots.

Each tree model offers a more or less detailed views of an RNA structure. Given the tree representations of two RNA secondary structure, one comparison way is based on the computation of the edit distance between the trees while the other focus on the alignment of the trees using the score of the alignment as a measure of the distance between the trees. Popular tools for optimal alignment of RNA secondary structures include RNAdistance [6] and RNAforester [8] etc. RNAdistance compares RNA secondary structures based on tree edit distance measure, while RNAforester computes the pairwise or multiple alignment of structures based on tree alignment measure. Hofacker [9] measured RNA secondary structures in terms of the base pairing probability matrices computed by McCaskill's partition function algorithm [10]. The popular tool based matrix of base pairing probabilities is RNApdist, which was implemented as part of the Vienna RNA package.

Because the above methods rely on dynamic programming algorithms, they are computation-intensive even if the pseudoknots are ignored. For example, the Sankoff's algorithm [11] simultaneously allows the structure prediction and alignment problem with *O*(*n*^4^) in memory and *O*(*n*^6^) in time for two RNA sequences of lengthn. So these algorithms are still impractical for long RNA sequences. Recently some comparison algorithms without aligning them are proposed. Kin [12] gave a kernel method based on Stochastic Context Free Grammar (SCFG).

The graphical representations of biosequences (protein, DNA and RNA) could be out of the mainstream but a new research view and tool to understand and analyze such biosequences. M.Randic [13] reviewed the sufficient materials on related topics of graphical representations of protein, DNA and the secondary structure of RNA. Inspired by several graphical representations of DNA sequences [14-18], some researchers have proposed 2D, 3D or 4D graphical approaches for the representations of RNA secondary structure and then derive some numerical invariants and different graphical measures from graphs to compare RNA secondary structures [19-32].

In [19-29], eight symbols of the unpaired bases A, C, G, U and paired bases A′, C′, G′, U′ were used to code RNA secondary structures as graphical representations. In [31], the representations of eight letters have been demonstrated to be approximate and have some loss of information. In [32], 12 symbols have been used to represent RNA secondary structure without loss of information, in which the key is to discriminate between the first and the second base of a hydrogen bond for the paired bases.

In this paper, motivated by DV curve representation of DNA sequences [33,34], we propose a novel triple vector curve representation of RNA secondary structure. With this novel representation, a new RNA secondary structure similarity measure based on wavelet analysis is designed, which can simultaneously focus on the local structure and global structure. To evaluate our algorithms, we take the classification of non-coding RNA and RNA mutation as examples to compare to the two popular tools of RNAdistance and RNApdist.

## Results and discussion

### Similarities/dissimilarities among non-coding RNA from different families

We performed the experiments on 100 RNA sequences to test the ability to distinguish non-coding RNA families. We randomly chose 25 sequences from each of the four RNA classes (5S rRNA, miRNA, RNaseP arch and tRNA) in RFAM database.

Firstly, the secondary structures of the 100 RNA sequences are predicted by the Vienna RNA folding prediction package. Secondly, their characteristic representations are constructed according to the primary sequence and the predicted secondary structure. Thirdly, the TV-Curves can be obtained based on their characteristic representations. Then we computed the similarity between any two RNA among these 100 RNA sequences by the proposed multi-scale similarity measure algorithm based on TV-Curve. Furthermore, all the similarity values are arranged into a similarity matrix. For validation of our algorithm, we computed the distance matrixes using RNApdist and RNAdistance tools respectively.

For the comparison of our multi-scale similarity measure with the popular RNA comparison tools, the validation index used here is Hubert’s statistic [35]. Let *X* and *Y* be *n×n* matrices, where *X*(*i,j*) indicates the observed similarity coefficient between the RNA *i* and *j*, and *Y*(*i,j*) represents the ground information defined as follows:

$$Y\left(i,j\right)=\{\begin{array}{l} 1,\;\text{if}\;\;\text{RNA}\;\;i\;\text{and}\;j\;\;\text{are}\;\;\text{in}\;\;\text{the}\;\;\text{same}\;\;\text{family}\\ 0,\;\text{otherwise} \end{array}$$

If *X*(*i,j*) indicates the observed distance between RNA *i* and *j*, then *Y*(*i,j*) is defined as

$$Y\left(i,j\right)=\{\begin{array}{l} 0,\;\text{if RNA}\;i\;\text{and}\;j\;\text{are in the same family}\\ 1,\;\text{otherwise} \end{array}$$

The Hubert's statistic represents the correlation between the matrices *X* and *Y* , which is defined as follows:

$$\begin{array}{c} H=\frac{2}{n\left(n-1\right)}\sum_{i=1}^{n-1}\sum_{j=i+1}^{n}\left(\frac{X\left(i,j\right)-\overset{―}{X}}{\sigma_{X}}\right)\left(\frac{Y\left(i,j\right)-\overset{―}{Y}}{\sigma_{Y}}\right)\\ =\frac{\sum_{i=1}^{n-1}\sum_{j=i+1}^{n}\left(X\left(i,j\right)-\overset{―}{X}\right)\left(Y\left(i,j\right)-\overset{―}{Y}\right)}{\sqrt{\sum_{i=1}^{n-1}\sum_{j=i+1}^{n}{\left(X\left(i,j\right)-\overset{―}{X}\right)}^{2}}\sqrt{\sum_{i=1}^{n-1}\sum_{j=i+1}^{n}{\left(Y\left(i,j\right)-\overset{―}{Y}\right)}^{2}}} \end{array}$$

where $\overset{―}{X}$ and $\overset{―}{Y}$ denote the means of the matrices of *X* and *Y*. The larger absolute value of H indicates the better coherence between the similarity matrix *X* and the ground matrix *Y*. The value of H can be used to estimate the quality of the similarity measure.

The Hubert's statistic for different similarity matrixes are shown in Table 1. The Hubert statistic of RNApdist and RNAdistance are 0.4095 and 0.1156 respectively.

**Table 1:** The Hubert statistic comparison for different similarity matrixes for 100 noncoding RNAs

**Method**	**RNApdist**	**RNAdistance**	**Multi-scale similarity based on TV-Curve**
Hubert statistic	0.4095	0.1156	0.7205

However, the Hubert's statistic of our proposed multi-scale similarity measure based on our algorithm is 0.7205. Obviously, our similarity measure is more closer to the real data compared with RNApdist and RNAdistance.

**Figure 1:**
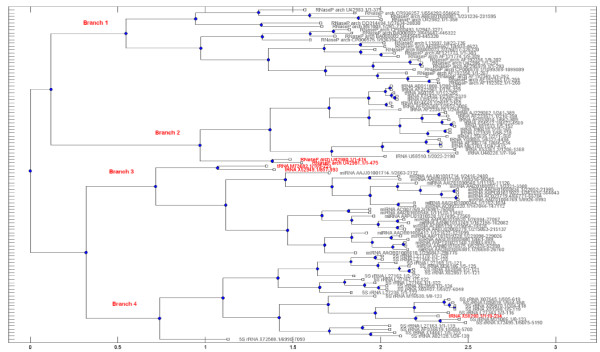
The Phylogenetic tree by multi-scale RNA comparison based on RNA triple vector curve representation using Unweighted Pair Group Method with Arithmetic Mean (UPGMA) for the four RNA classes (5S rRNA, miRNA, RNaseP arch and tRNA).

In addition, to further compare the performance of our method with the RNApdist and RNAdistance, we reconstructed three phylogenetic trees (see Figure 1, Additional file 1: Figure S2 and Figure S3) using Unweighted Pair Group Method with Arithmetic Mean (UPGMA) [36] according to the pairwise similarities of the RNA sequences obtained by multi-scale similarity measure algorithm based on TV-Curve, RNApdist and RNAdistance, respectively.

Obviously, compared Additional file 1: Figure S1 and Figure S2 with Figure 1, the phylogenetic tree based on our proposed measure presents clearly four branches. The four branches of Figure 1 can be regarded as the classification of the 100 RNA sequences, where branch 1 with 23 RNaseP_archs, branch 2 with 22 tRNAs and 2 RNaseP_archs, branch 3 with 25 miRNA and 2 tRNA, and branch 4 with 25 5S_rRNAs and 1 tRNAs. It is easy to obtain the false percentage is 5%. Moreover, the 2 RNaseP_archs in branch 2 and 2 tRNA in branch 3 are both isolated from the 22 tRNAs and 25 miRNA respectively. The distinguished performance of our proposed method is better than the popular RNA comparison RNApdist and RNAdistance tools.

### Similarities/dissimilarities among the RNA secondary structures of nine virus

**Figure 2:**
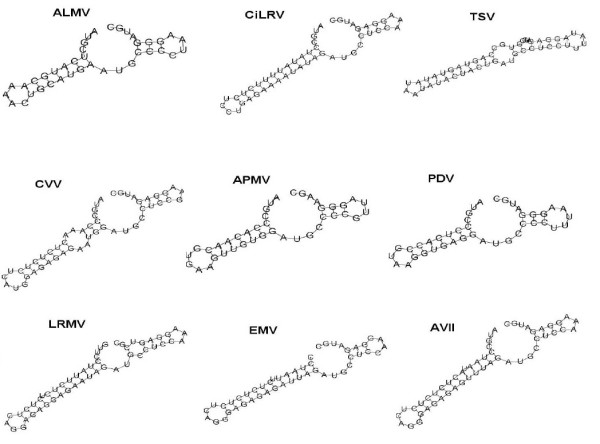
The secondary structures at the 3'-terminus of RNA 3 of nine viruses: Alfalfa Mosaic Virus (ALMV), Citrus Leaf Rugose Virus (CiLRV), Tobacco Streak Virus (TSV), Citrus Variegation Virus (CVV), Apple Mosaic Virus (APMV), Prune Dwarf Ilarvirus (PDV), Lilac Ring Mottle Virus (LRMV), Elm Mottle Virus (EMV) and asparagus virus II (AVII).

**Figure 3:**
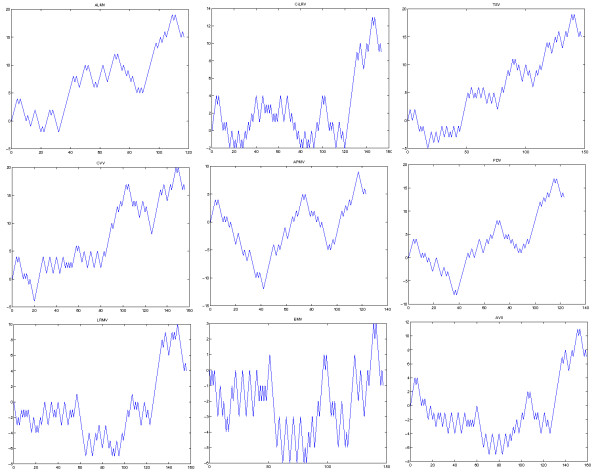
The TV-Curves at the 3'-terminus of RNA 3 of nine viruses: Alfalfa Mosaic Virus (ALMV), Citrus Leaf Rugose Virus (CiLRV), Tobacco Streak Virus (TSV), Citrus Variegation Virus (CVV), Apple Mosaic Virus (APMV), Prune Dwarf Ilarvirus (PDV), Lilac Ring Mottle Virus (LRMV), Elm Mottle Virus (EMV) and Asparagus Virus II (AVII).

To further illustrate the utility of our approach for the subtle structure comparison, we examine similarities /dissimilarities of a set of relatively similar RNA secondary structures at the 3’-terminus of nine different viruses. The nine virus include alfalfa mosaic virus (ALMV), citrus leaf rugose virus (CiLRV), tobacco streak virus (TSV), citrus variegation virus (CVV), apple mosaic virus (APMV), prune dwarf ilarvirus (PDV), lilac ring mottle virus (LRMV), elm mottle virus (EMV) and asparagus virus II (AVII). The predicted corresponding secondary structures and corresponding TV-Curves are given in Figure 2 and Figure 3. Their similarity matrix obtained by multi-scale similarity measure is shown in Table 2.

**Table 2:** **The similarity matrix for the secondary structures at the 3'-terminus belonging to nine viruses of Figure **2** by multi-scale RNA comparison based on RNA triple vector curve representation.**

**Species**	**ALMV**	**CiLRV**	**TSV**	**CVV**	**APMV**	**LRMV**	**PDV**	**EMV**	**AVII**
ALMV	1.0000	0.2596	0.2300	0.1281	0.1638	0.2606	0.4545	0.2688	0.3770
CiLRV		1.0000	0.3259	0.4983	0.2678	0.5929	0.2007	0.4241	0.4337
TSV			1.0000	0.3828	0.2869	0.2888	0.3054	0.1652	0.1443
CVV				1.0000	0.3947	0.6029	0.2755	0.3217	0.5566
APMV					1.0000	0.1912	0.7407	0.3245	0.1886
LRMV						1.0000	0.1734	0.4963	0.6387
PDV							1.0000	0.3033	0.1187
EMV								1.0000	0.4248
AVII									1.0000

The maximal similarity is 1.0000.

**Figure 4:**
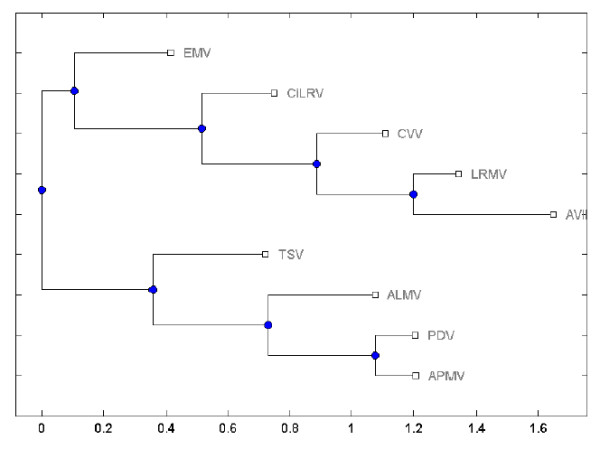
The phylogenetic tree for nine virus by multi-scale RNA comparison based on RNA triple vector curve representation using Unweighted Pair Group Method with Arithmetic Mean (UPGMA).

To further present our result, we constructed a phylogenetic tree with UPGA algorithm for the nine virus using the multi-scale similarity measure based on TV-Curves shown in Figure 4.

Observing Table 2 and Figure 4, we find the most similar species pairs are (PDV,APMV) and (AVII, LRMV), and the next similar species pairs are (ALMV,PDV),(ALMV,APMV), (AVII, CVV) and (CVV,LRMV). The results are analogous to the difference of the secondary structures in Figure 2, which show that our approach also present the better performance for similar secondary structure comparison.

### RNA mutation analysis

**Figure 5:**
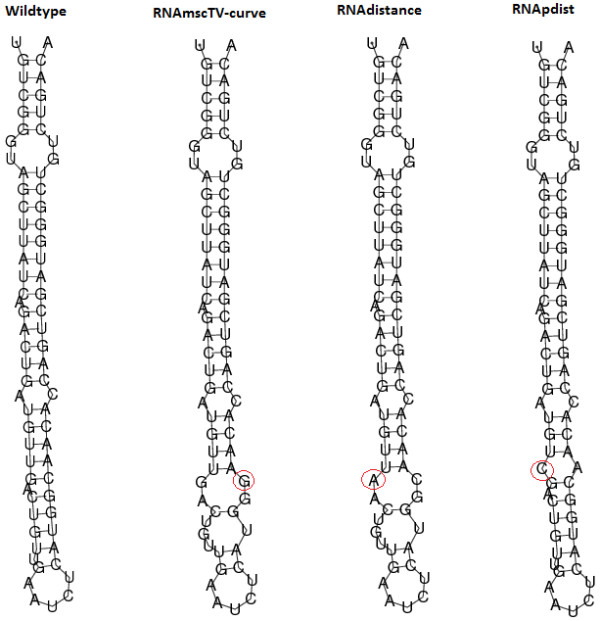
Largest structure mutation for microRNA miR-21 precursor sequences using multi-scale RNA comparison based on RNA triple vector curve representation (RNAmscTV-Curve), RNAdistance and RNApdist.

**Figure 6:**
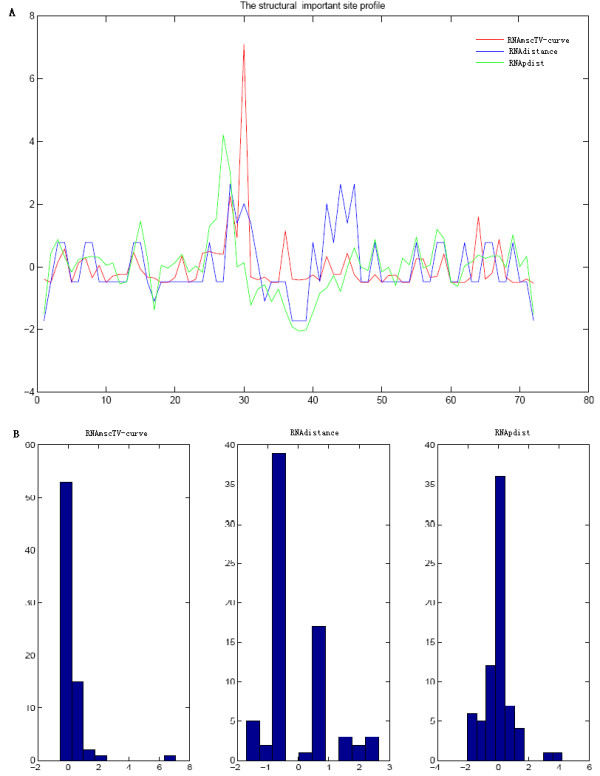
Structural deleteriousness profiles analysis (A) Comparison of Structural deleteriousness profiles for microRNA miR-21 precursor sequences between RNAmscTV-Curve, RNAdistance and RNApdist; (B) Histograms of Structural deleteriousness profile for microRNA miR-21 precursor sequences based on RNAmsctriv, RNAdistance and RNApdist.

Mutations in RNA structure may lead to impair functions resulting in diseases, but RNA structure mutations could be beneficial in some situation. Consequently, it is very important to search the most significant point mutation. Our proposed method is very efficient to find the significant point mutation compared with the popular RNA mutation analysis tool: RDMAS [1]. RDMAS is a web server for evaluating structural deleteriousness of single nucleotide mutation in RNA genes. We evaluate single nucleotide structure mutation microRNA miR-21 precursor based on TV-Curve representation and compared to RDMAS tool. Meanwhile compared to RNAdistance and RNApdist, we predict the most significant point mutation shown in Figure 5. In RDMAS, the maximum difference in structures between the wild-type and the possible mutation at each position are extracted into a structural deleteriousness profile. We compare the deleteriousness profiles and their histograms between our method, RNAdistance and RNApdist (Figure 6A). As shown in Figure 6B, it is obviously to see that our method can find more significant structural mutations compared with RNAdistance and RNApdist.

Additionally, in order to further validate the efficiency of our method, we test the 21 rRNA fragments of the thermus thermophilus from Ribosomal data-set in [37] compared with RNAdistance and RNApdist. The labels and sequences are listed in Table S1 (See Additional file 2: Table S1). In Table S2 (See Additional file 2: Table S2), the most significant mutation position and type are listed for RNAmscTV-curve (RNA multi-scale RNA comparison based on RNA triple vector curve), RNAdistance and RNApdist. Out of the 21 RNA sequences in the data set, 3 fragments (E_(68),A_(588–651),A_(1113–1187), A_(240–286)) produced the same most significant mutation as RNAdistance and RNApdist. Our proposed structure for the rest fragments are more different than the structure with the largest RNAdistance and RNApdist but it is non-obvious to determine which one of them is more significant. Both of the mutations alter the structure with respect to the original structure. The results in Figure S3 (See Additional file 1: Figure S3) provide further evidence that our method can capture more significant and subtle structure mutation.

## Conclusion

In this paper, we provide a better visualization and analysis tool TV-Curve for RNA to indicate the information of sequence and secondary structure especially for long RNA. Additionally, based on TV-Curves representation of RNA, a multi-scale similarity measure for RNA comparison is proposed, which can capture the local and global difference between the information of sequence and structure of RNA. Compared with the popular RNA comparison approaches, the proposed method is evaluated to be outstanding and effective. But as we know, the native secondary structure of a RNA is often a suboptimal structure not the predicted structure with minimum free energy (MFE) due to limitations of thermodynamic models. The structural similarity measurement using multiple predicted suboptimal structures is still a challenge. In the further research, we will focus on how to measure the structural similarity to integrate multiple structures with different energy levels.

## Method

### The TV-Curve representation of RNA secondary structure

In this section, we describe the construction of TV-Curve of the secondary structure of RNA. Firstly, we give the characteristic representation of RNA based on the primary and secondary structure of RNA.

### The characteristic representation of RNA secondary structure

In [15], Liao proposed a characteristic representation of RNA secondary structure, which both include the primary structure and secondary structure. This representation was based on eight symbols, the four A, C, G, U for the four nucleotide bases (adenine, cytosine, guanine and uracil, respectively) and four A', C', G', U' for the same bases if paired by hydrogen bonds. In the primary structure of RNA, let A', U', G' and C' denote A, U, G and C in the base pair *A-U, G-C* or *G-U*, respectively. A characteristic sequence of the secondary structure can be obtained. The RNA secondary structure is predicted by the Vienna RNA folding prediction package [38].

Combining the information of the sequence and secondary structure, we give the corresponding characteristic sequence of the secondary structure of tRNA **(*U48228.1/7-166*)** in the following:***>tRNA (U48228.1/7-166)****CAAU'C'U'UAA'CG'A'U'G'G'AUG'U'C'U'U'GG'U'U'CC'UAUAG'CG'A'U'GA'A'G'G'C'C'G'C'A'G'C'A'AAGU'G'C'GAUAU'G'C'A'AU'GAAAA'AU'G'C'A'AUUACU'G'U'G'AAU'C'A'U'C'AG'A'A'U'G'C'UGAA'U'G'U'AAA'CUAUAC'C'A'U'A'UUUACCCUU'A'U'G'G'GCAAAU'UAA'C'G'U'GG'U'A'U'U'C'CU'ACA'G'A'AA.*

### Construction of TV-Curve

**Figure 7:**
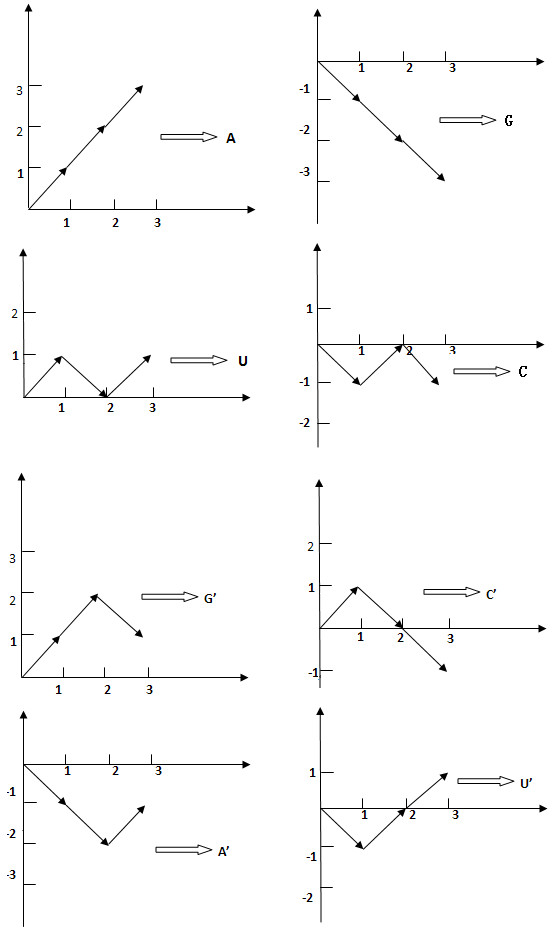
TV-Curve Representation: The numerical representations of four unpaired nucleotides (A, T, C and G) and four paired nucleotides (A', U', G' and C') of TV-Curve.

In this subsection, the construction of TV-Curve is given. As shown in Figure 7, each alphabet of A, T, C,G, A', U', G' and C' is represented by three vectors as follows:

(1) $$\begin{array}{l} (1,1),(1,1),(1,1)\Rightarrow A,\;(1,-1),(1,-1),(1,1)\Rightarrow A^{\prime }\\ (1,1),(1,-1),(1,1)\Rightarrow U,\;(1,-1),(1,1),(1,1)\Rightarrow U^{\prime }\\ (1,-1),(1,-1),(1,-1)\Rightarrow G,\;(1,1),(1,1),(1,-1)\Rightarrow G^{\prime }\\ (1,-1),(1,1),(1,-1)\Rightarrow C,\;(1,1),(1,-1),(1,-1)\Rightarrow C^{\prime } \end{array}$$

TV-Curve can be obtained by connecting all the vectors one by one. We give two corresponding mathematical models of TV-Curve. Denote a characteristic sequence of RNA as S = *S*_1_*S*_2_ ⋯ *S*_*n*_ where *S*_*i*_ ∈ {A, T, C, G, A ', T ', C ', G '} and n is the length of this characteristic sequence. Define the corresponding TV-Curve as

$$\left(X,Y\right),X=x_{0}x_{1}\cdots x_{3n},Y{\text{=y}}_{0}y_{1}\cdots y_{3n}\text{,}$$

which can be obtained by the following formulas:

(2) $$\begin{array}{l} A;\;\text{if}\;\left\{\begin{array}{l} y_{3i}-y_{3i-1}=1,y_{3i-1}-y_{3i-2}=1\\ y_{3i-2}-y_{3i-3}=1 \end{array}\right)\\ U;\;\text{if}\;\left\{\begin{array}{l} y_{3i}-y_{3i-1}=1,y_{3i-1}-y_{3i-2}=-1\\ y_{3i-2}-y_{3i-3}=1 \end{array}\right)\\ G;\;\text{if}\;\left\{\begin{array}{l} y_{3i}-y_{3i-1}=-1,y_{3i-1}-y_{3i-2}=-1\\ y_{3i-2}-y_{3i-3}=-1 \end{array}\right)\\ C;\;\text{if}\;\left\{\begin{array}{l} y_{3i}-y_{3i-1}=-1\\ y_{3i-1}-y_{3i-2}=1\\ y_{3i-2}-y_{3i-3}=-1 \end{array}\right)\\ A^{\prime };\;\text{if}\;\left\{\begin{array}{l} y_{3i}-y_{3i-1}=-1,y_{3i-1}-y_{3i-2}=-1\\ y_{3i-2}-y_{3i-3}=1 \end{array}\right)\\ U^{\prime };\;\text{if}\;\left\{\begin{array}{l} y_{3i}-y_{3i-1}=-1,y_{3i-1}-y_{3i-2}=1\\ y_{3i-2}-y_{3i-3}=1 \end{array}\right)\\ G^{\prime };\;\text{if}\;\left\{\begin{array}{l} y_{3i}-y_{3i-1}=1,y_{3i-1}-y_{3i-2}=1\\ y_{3i-2}-y_{3i-3}=-1 \end{array}\right)\\ C^{\prime };\;\text{if}\;\left\{\begin{array}{l} y_{3i}-y_{3i-1}=1,y_{3i-1}-y_{3i-2}=-1\\ y_{3i-2}-y_{3i-3}=-1 \end{array}\right)\\ \;i=1,2,\ldots ,n. \end{array}$$

For a given TV-Curve of RNA, we can retrieve its characteristic representation from equation (2).

**Figure 8:**
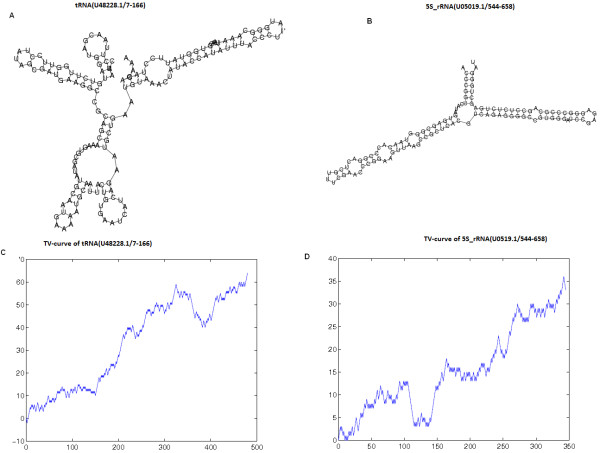
**TV-Curve representation: (A) The secondary structure of tRNA *(U48228.1/7-166); *(B) The secondary structure of 5S _rRNA *(U05019.1/544-658); *(C) The TV-Curve of tRNA *(U48228.1/7-166); *(D) The TV-Curve of 5S _rRNA *(U05019.1/544-658).***

For example, we give the secondary structures and the corresponding TV-Curves of tRNA (***U48228.1/7-166)*** and 5S_rRNA ***(U05019.1/544-658)*** from the equation (1) and (2) (See Figure 8). From Figure 8C-D, it is very easy to identify the difference between 5S_rRNA ***(U05019.1/544-658)*** and tRNA ***(U48228.1/7-166).*** In order to further prove the difference of the two TV-Curves, the fractal dimensions of the TV-Curves of 5S_rRNA ***(U05019.1/544-658)*** and tRNA (***U48228.1/7-166***) is 0.6404 and 0.5958.

The TV-Curve is a good visualization method to represent the information of the primary and secondary structure of a RNA molecular especially for long RNA sequence. In addition, the TV-Curve is a numerical representation of RNA, which provides another view to understand RNA. From the above construction, some properties of TV-Curve can be easily obtained:

(1). TV-Curve extends 3 units along X-axis to represent each unpaired nucleotide (A, T, C G) and paired nucleotide (A', T', C' G').

(2). From TV-Curve, one can immediately grasp the information about RNA sequence and structure information. From a given TV-Curve, we can obtain its unique sequence and secondary structure representation. Moreover, for a given RNA sequence and structure, there is a unique TV-Curve representation. The correspondence between TV-Curves and the RNA information of sequences and secondary structures is one to one and no loss of information. If one wants to know whether the i-th nucleotide in RNA sequence is paired, only need to examine the difference between the values at (3i-2) and (3i-3) of TV-Curve. If *y*_3*i* − 2_ − *y*_3*i* − 3_ = 1, the i-th nucleotide is paired. If *y*_3*i* − 2_ − *y*_3*i* − 3_ = − 1, the i-th nucleotide is unpaired.

(3). The X-axis end point *x*_*end*_ of the TV-Curve indicates the length of RNA sequence n, i.e. *n* = *x*_*end*_/3.

### Multi-scale similarity measure based on TV-Curves

In this section, based on TV-Curves of RNA, we propose a multi-scale similarity measure for RNA comparison in terms of the multi-scale property of wavelet transform.

We estimate RNA similarity using the weighted correlations in the wavelet domains at the different scales. The main characteristics of wavelet transforms are time-frequency localization and multi-resolution property. Wavelet can capture the global and local property of a signal synchronously and can focus on the any detail of a signal. In this sense wavelets are referred to as a mathematical microscope. In the following, we briefly introduce the discrete wavelet transform [39,40].

The wavelet transform relies on the wavelet function *ψ*(x) and the scaling function *∅ϕ*(*x*), which

satisfies the following two-scale relation:

$$\phi \left(x\right)=\sqrt{2}\sum_{n}h_{n}\phi \left(2x-n\right)\text{,}$$

Where {*h*_*n*_} is a low-pass filter (scaling filter).

The associated wavelet function constructed using scaling function satisfies the following equation:

$$\psi \left(x\right)=\sqrt{2}\sum_{n}g_{n}\phi \left(2x-n\right)\text{,}$$

Where {*g*_*n*_} is a high-pass filter (wavelet filter)

Given a signal s with length N, the wavelet transform consists of *log*_*2*_*N* levels at most. The wavelet decomposition of the signal s analyzed at level one is provided with two sets of coefficients: approximation coefficients *cA*_*1*_, and detail coefficients *cD*_1_. *cA*_1_ is obtained by convolving s with the low-pass filter and then is downsampled (keep the even index elements) for approximation, and *cD*_*1*_ is also obtained by the high-pass filter and then is downsampled for detail.

**Figure 9:**
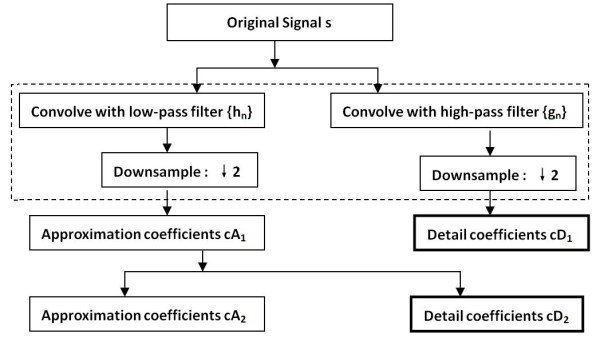
The flow chart of wavelet decomposition.

The wavelet decomposition at level two analyzed the approximation coefficients *cA*_*1*_ in two sets using the same scheme, replacing s by *cA*_*1*_, and producing the approximation coefficients *cA*_*2*_ and detail coefficients *cD*_*2*_. The wavelet decomposition of the signal s analyzed at level j has the approximation coefficients *cA*_*j*_ and detailed coefficients *cDj*, …, and *cD*_*1*_ at different level. In Figure 9, the flow chart of wavelet decomposition is given.

For any signal s denote *cA*_0_ = {*c*_*k*_^0^} = *s*. At level j, the corresponding approximation coefficient *cA*_*j*_ = {*c*_*k*_^*j*^} and detail coefficient *cD*_*j*_ = {*d*_*k*_^*j*^} can be fast computed by Mallat algorithm [39] as follows:

$$\left\{ \begin{array}{ccc} c_{k}^{j} & = & \sum_{l\in Z}h_{l-2k}c_{l}^{j-1},\\ d_{k}^{j} & = & \sum_{l\in Z}g_{l-2k}c_{l}^{j-1},\;k\in Z\text{.} \end{array}\right.$$

And if {*h*_*l*_}_*l*_ and {*g*_*l*_}_*l*_ are orthogonal, there is *g*_*l*_ = (−1)^*l*^*h*_1 − *l*_. While in the biorthogonal condition there are four filters (two group filters): decomposition filters {*h*_*l*_}_*l*_, {*g*_*l*_}_*l*_, reconstruction filters ${\left\{{\tilde{h}}_{l}\right\}}_{l},{\left\{{\tilde{g}}_{l}\right\}}_{l}$, where $g_{l}={\left(-1\right)}^{l}{\tilde{h}}_{1-l}$ and ${\tilde{g}}_{l}={\left(-1\right)}^{l}h_{1-l}$.

**Figure 10:**
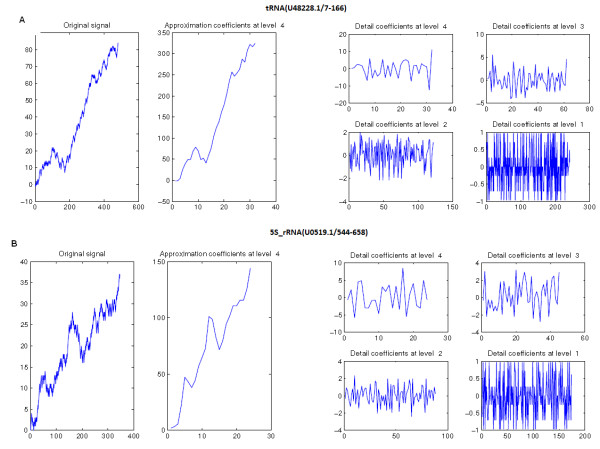
**The wavelet decomposition of the TV-Curves: (A) the wavelet Decomposition of tRNA (*U05019.1/544-658*) ; (B) the wavelet Decomposition of 5S_rRNA (*U05019.1/ 544–658*).**

We applied the wavelet decomposition to the TV-Curves of tRNA (***U05019.1/544-658***) and 5S_rRNA (***U05019.1/ 544–658***) (See Figure 10), which can help us to capture the local and global difference between them.

Based on the wavelet decomposition of TV-Curves of RNA sequences, we design a novel similarity measure for RNA comparison by the combination of Pearson correlation coefficient and multi-resolution feature of wavelet, which can capture the local and global similarity at the same time. For two given RNA TV-Curves *Y*_*1*_ and *Y*_*2*_, it is easy to extend they have the same length N using period extend or zero extend. The Pearson correlation between *Y*_*1*_ and *Y*_*2*_ is defined as:

$$P_{Y_{1},Y_{2}}=\frac{\sum_{i=1}^{N}(Y_{1}\left(i\right)-\overset{―}{Y_{1}})\left(Y_{2}\left(i\right)-\overset{―}{Y_{2}}\right)}{\sqrt{\sum_{i=1}^{N}\left(Y_{1},(,i,),-,\overset{―}{Y_{1}}\right){}^{2}}\sqrt{\sum_{i=1}^{N}\left(Y_{2},(,i,),-,\overset{―}{Y_{2}}\right){}^{2}}}$$

We firstly decompose the two TV-Curves *Y*_*1*_ and *Y*_*2*_ with L level wavelet transform. Here L=4. After the four level transform, we obtained the detail coefficients $\left\{cD_{4}^{Y_{i}},cD_{3}^{Y_{i}},cD_{2}^{Y_{i}},cD_{1}^{Y_{i}}\right\}$ and approximation coefficients $\left\{cA_{4}^{Y_{i}}\right\},i=1,2$. Then the Pearson correlation coefficients $P_{cA_{4}^{Y_{1}},cA_{4}^{Y_{2}}}$ and $\left\{P_{cD_{i}^{Y_{1}},cD_{i}^{Y_{2}}},i=1,\ldots ,4\right\}$ at different decomposition levels are calculated and the weighted sum is taken as the multi-scale similarity *S* between *Y*_*1*_ and *Y*_*2*_ using each level's resolution proportion as weight as follows:

$$S=\frac{2^{\left(L-1\right)/2}P_{cA_{4}^{Y_{1}},cA_{4}^{Y_{2}}}+\sum_{i=1}^{L}2^{\left(i-1\right)/2}P_{cD_{i}^{Y_{1}},cD_{i}^{Y_{2}}}}{2^{\left(L-1\right)/2}+\sum_{i=1}^{L}2^{\left(i-1\right)/2}}$$

## Abbreviations

TV-curve: Triple vector curve; RNAmscTV-curve: RNA multi-scale comparison based on Triple vector curve.

## Competing interests

The authors declare that they have no competing interests.

## Authors' contributions

LY formulated the mathematical model and drafted the original manuscript. DM revised the manuscript and consulted on the experiments. LYC conceived the study and revised the manuscript. All authors contributed to the design and writing of the manuscript. All authors read and approved the final manuscript.

## Supplementary Material

Additional file 1: Figure S1 The Phylogenetic tree by RNApdist using Unweighted Pair Group Method with Arithmetic Mean (UPGMA) for the four RNA classes (5S rRNA, miRNA, RNaseP arch and tRNA). **Figure S2:** The Phylogenetic tree by RNAdistance using Unweighted Pair Group Method with Arithmetic Mean (UPGMA) using Unweighted Pair Group Method with Arithmetic Mean (UPGMA) for the four RNA classes (5S rRNA, miRNA, RNaseP arch and tRNA). **Figure S3:** Largest structure mutation for 21 RNA Ribosomal sequences using RNAmscTV-Curve, RNAdistance and RNApdist. Click here for file

Additional file 2: Table S1 21 ribosomal RNA fragments of thermus thermophilus HB8. **Table S2:** The mutations with the largest difference from the wild types of 21 ribosomal RNA fragments using RNAmscTV-Curve, RNAdistance and RNApdist. Click here for file
