# Supplementary material for: Multi-scale RNA comparison based on RNA triple vector curve representation
Source: BMC Bioinformatics. 2012 Oct 30;13:280. doi: 10.1186/1471-2105-13-280 (PMC3599440; doi:10.1186/1471-2105-13-280)
Supplement: Additional file 1: Figure S1 — The Phylogenetic tree by RNApdist using Unweighted Pair Group Method with Arithmetic Mean (UPGMA) for the four RNA classes (5S rRNA, miRNA, RNaseP arch and tRNA). Figure S2: The Phylogenetic tree by RNAdistance using Unweighted Pair Group Method with Arithmetic Mean (UPGMA) using Unweighted Pair Group Method with Arithmetic Mean (UPGMA) for the four RNA classes (5S rRNA, miRNA, RNaseP arch and tRNA). Figure S3: Largest structure mutation for 21 RNA Ribosomal sequences using RNAmscTV-Curve, RNAdistance and RNApdist. [file 1471-2105-13-280-S1.pdf]

Figure S1: The Phylogenetic tree by RNApdist using Unweighted Pair Group Method with Arithmetic Mean (UPGMA) for the four RNA classes (5S rRNA, miRNA, RNaseP arch and tRNA).

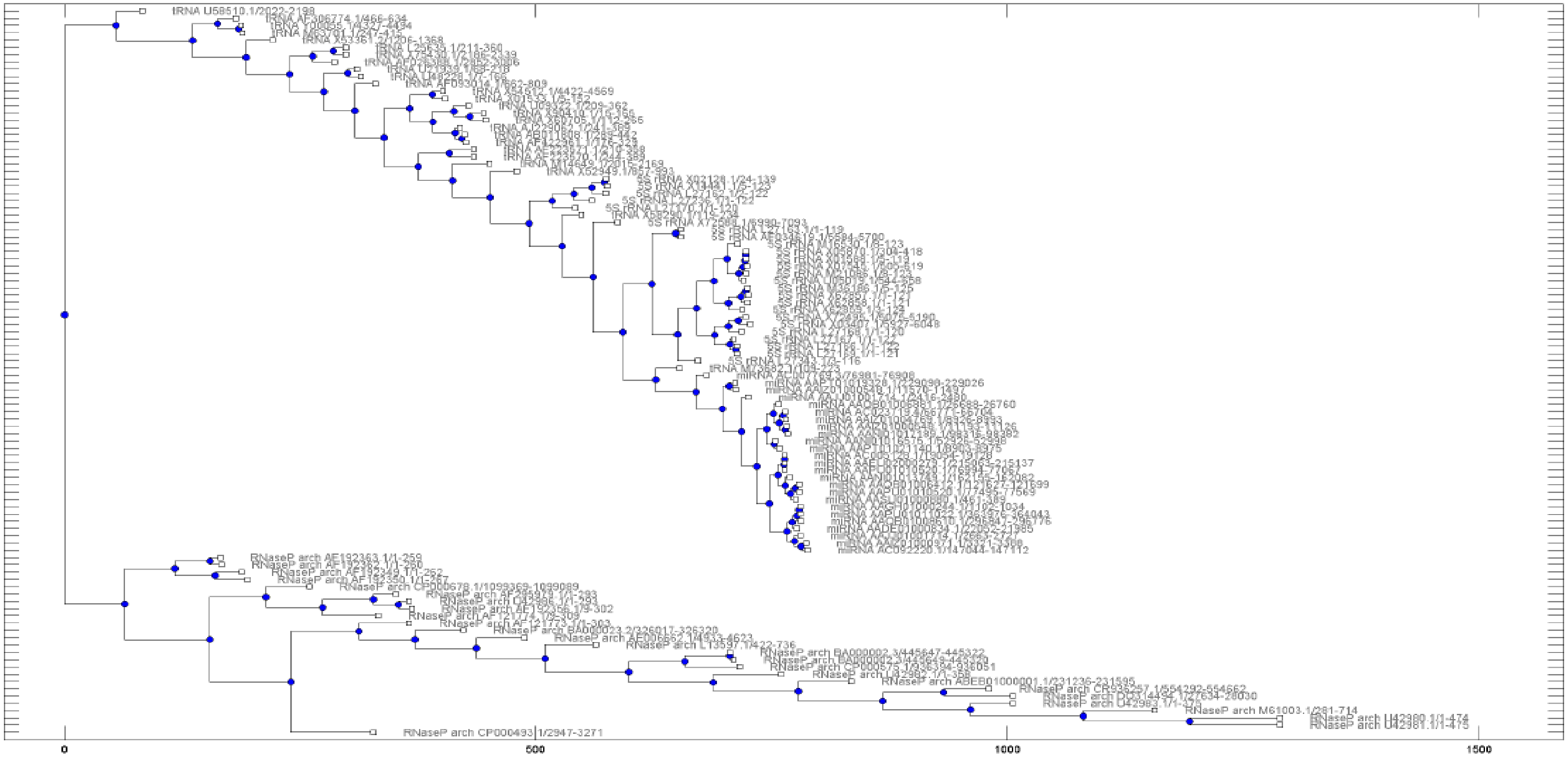

Figure S2: The Phylogenetic tree by RNAdistance using Unweighted Pair Group Method with Arithmetic Mean (UPGMA) using Unweighted Pair Group Method with Arithmetic Mean (UPGMA) for the four RNA classes (5S rRNA, miRNA, RNaseP arch and tRNA).

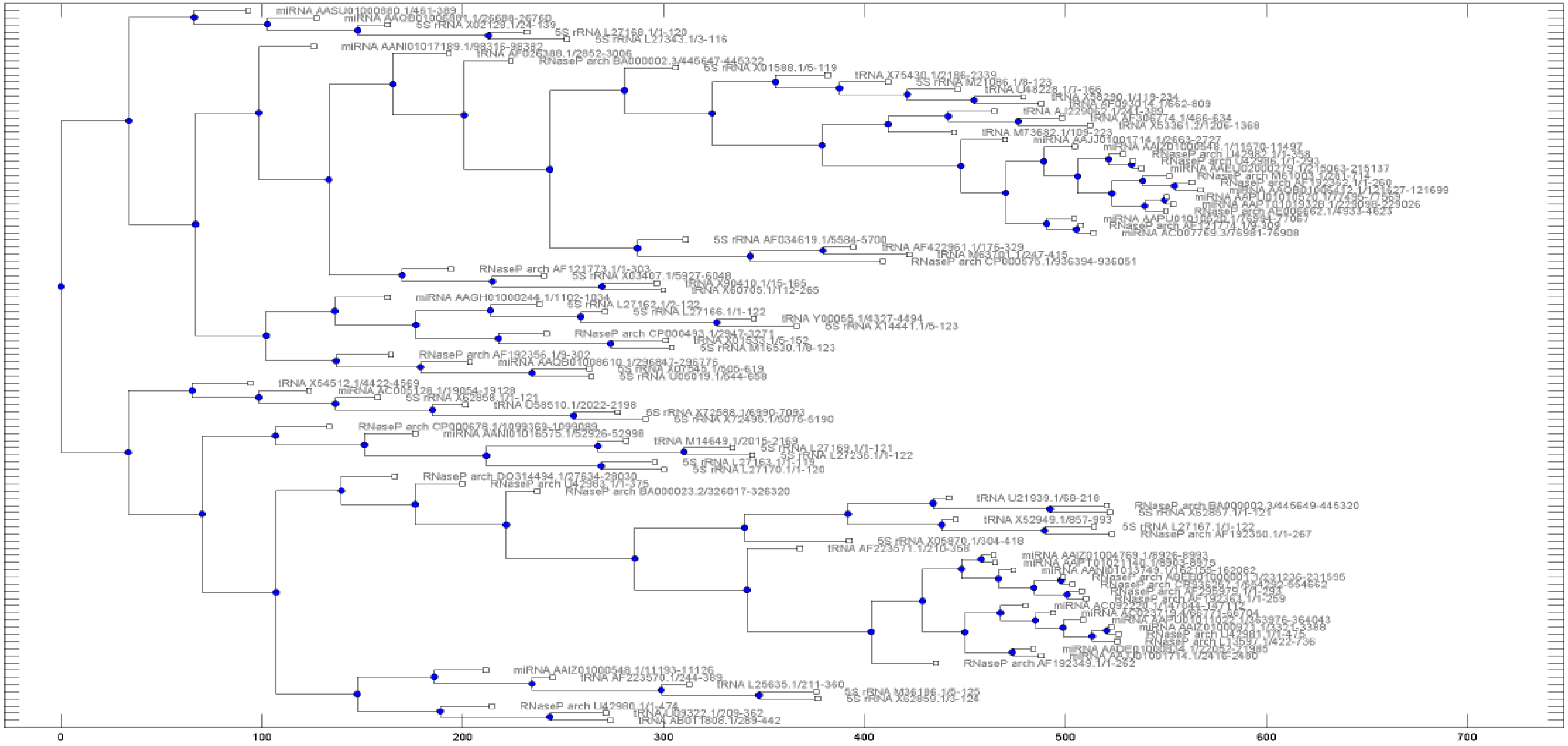

Figure S3: Largest structure mutation for 21 RNA Ribosomal sequences using RNAmscTV-Curve, RNAdistance and RNApdist

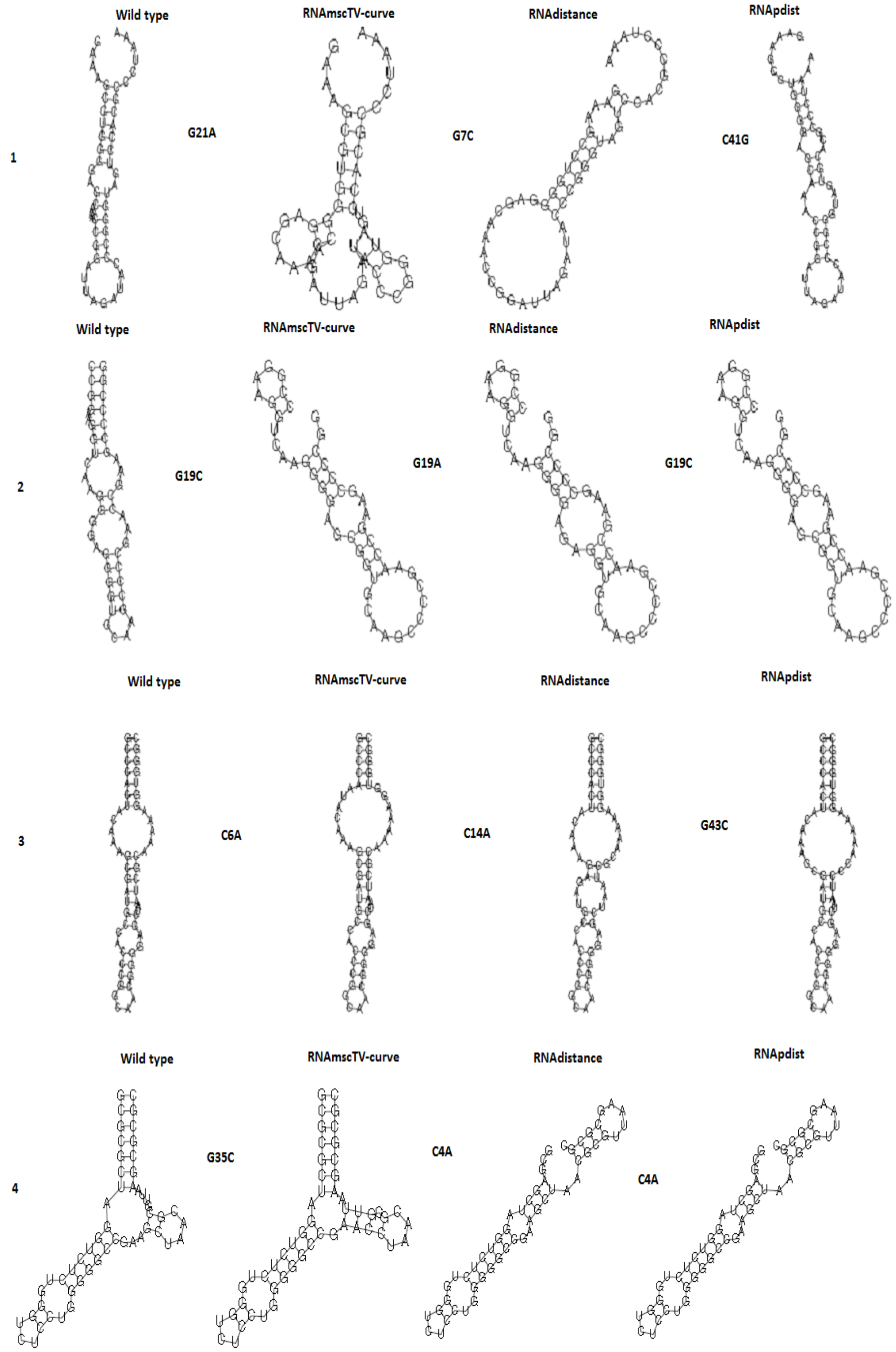

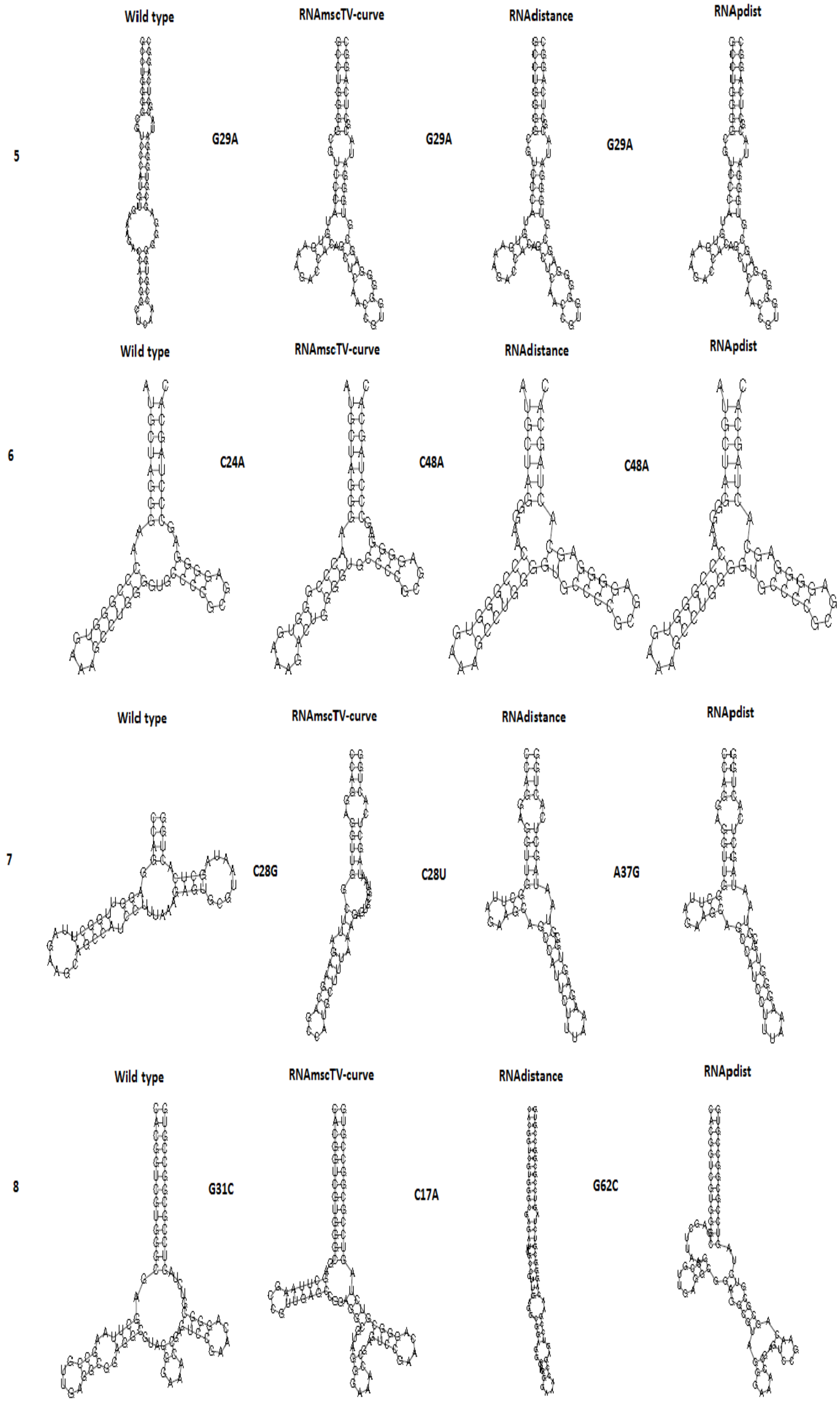
Wild type
RNAmscTV-curve
RNAdistance
RNApdist
5
G29A
G29A
G29A
Wild type
RNAmscTV-curve
RNAdistance
RNApdist
6
C24A
C48A
C48A
Wild type
RNAmscTV-curve
RNAdistance
RNApdist
7
C28G
C28U
A37G
Wild type
RNAmscTV-curve
RNAdistance
RNApdist
8
G31C
C17A
G62C

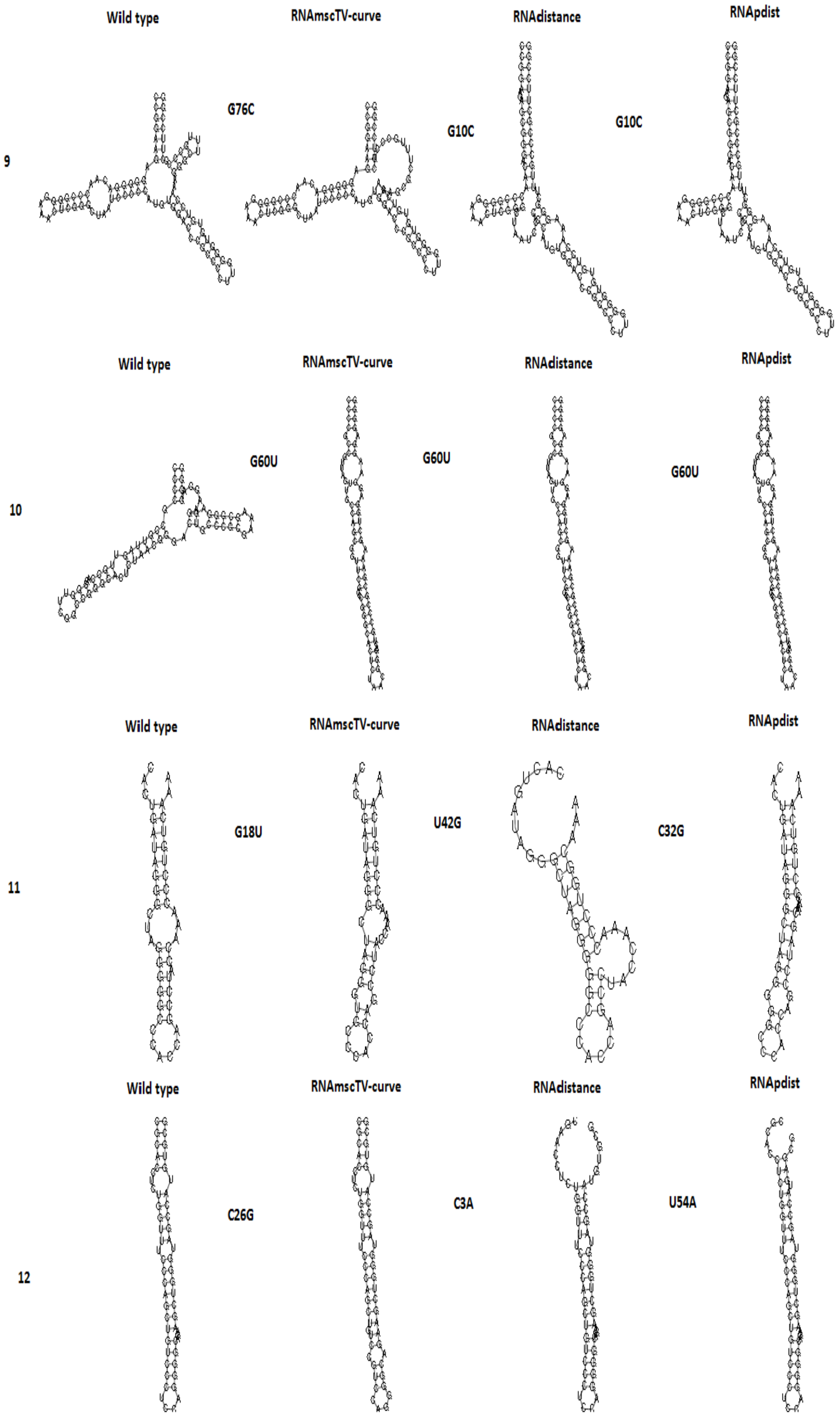
Wild type
RNAmscTV-curve
RNAdistance
RNApdist
9
G76C
G10C
G10C
Wild type
RNAmscTV-curve
RNAdistance
RNApdist
10
G60U
G60U
G60U
Wild type
RNAmscTV-curve
RNAdistance
RNApdist
11
G18U
U42G
C32G
Wild type
RNAmscTV-curve
RNAdistance
RNApdist
12
C26G
C3A
U54A

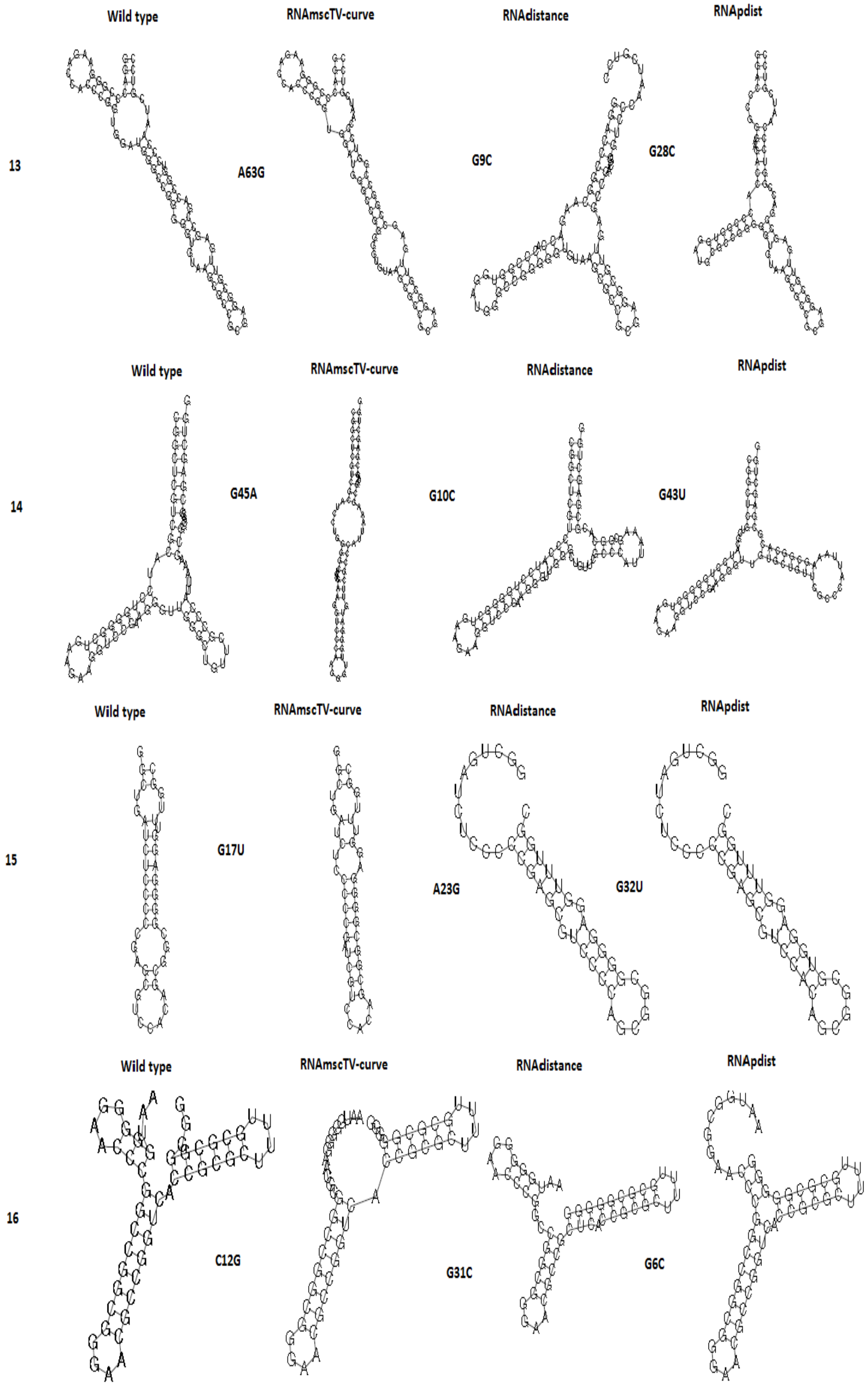
Wild type
RNAmscTV-curve
RNAdistance
RNApdist
13
A63G
G9C
G28C
Wild type
RNAmscTV-curve
RNAdistance
RNApdist
14
G45A
G10C
G43U
Wild type
RNAmscTV-curve
RNAdistance
RNApdist
15
G17U
A23G
G32U
Wild type
RNAmscTV-curve
RNAdistance
RNApdist
16
C12G
G31C
G6C

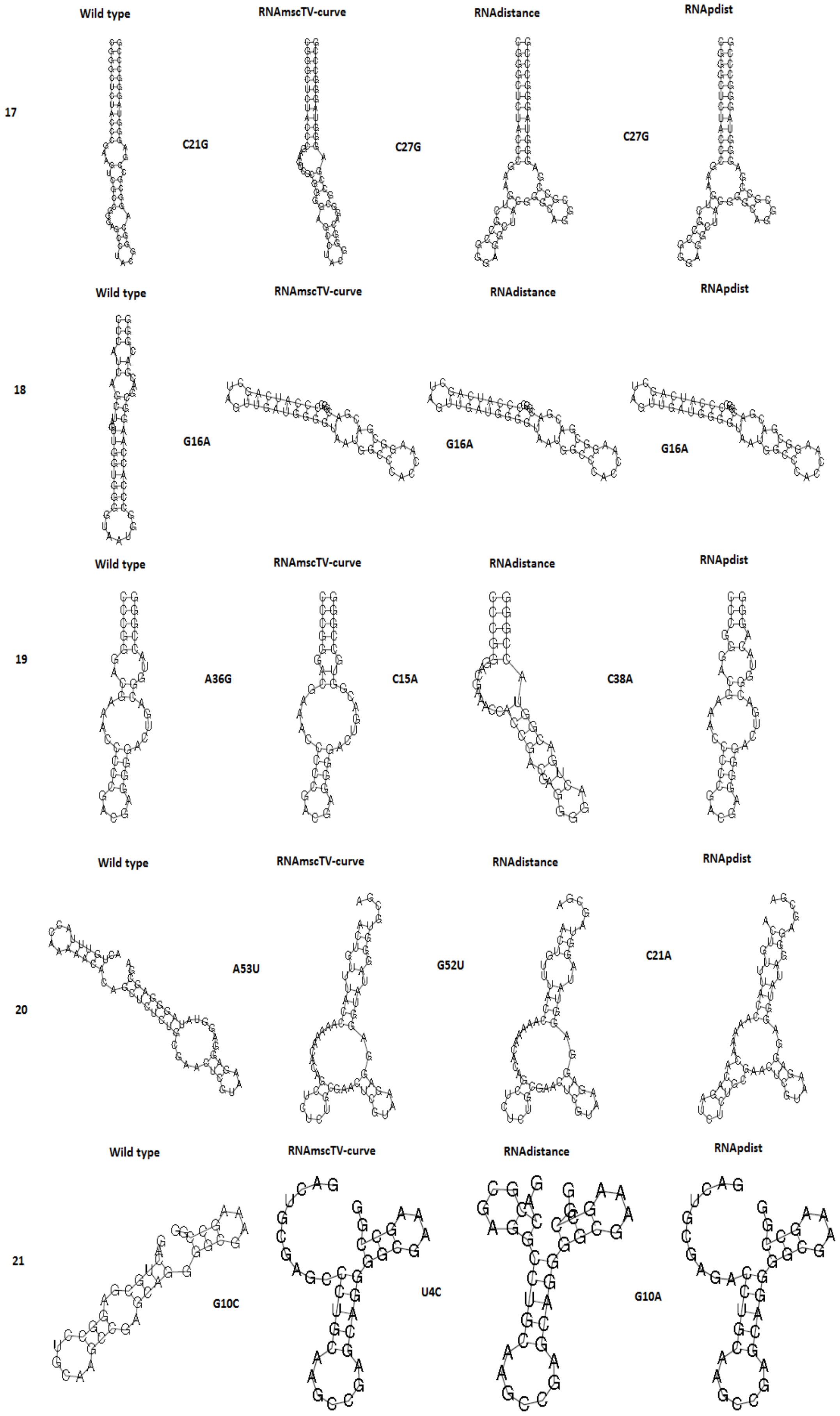
17
Wild type
C21G
RNAmscTV-curve
C27G
RNAdistance
C27G
RNApdist
18
Wild type
G16A
RNAmscTV-curve
G16A
RNAdistance
G16A
RNApdist
19
Wild type
A36G
RNAmscTV-curve
C15A
RNAdistance
C38A
RNApdist
20
Wild type
A53U
RNAmscTV-curve
G52U
RNAdistance
C21A
RNApdist
21
Wild type
G10C
RNAmscTV-curve
U4C
RNAdistance
G10A
RNApdist
