# Supplementary material for: Multi-scale RNA comparison based on RNA triple vector curve representation
Source: BMC Bioinformatics. 2012 Oct 30;13:280. doi: 10.1186/1471-2105-13-280 (PMC3599440; doi:10.1186/1471-2105-13-280)
Supplement: Additional file 2: Table S1 — 21 ribosomal RNA fragments of thermus thermophilus HB8. Table S2: The mutations with the largest difference from the wild types of 21 ribosomal RNA fragments using RNAmscTV-Curve, RNAdistance and RNApdist. [file 1471-2105-13-280-S2.pdf]

Table S1: 21 ribosomal RNA fragments of *thermus thermophilus* HB8

Index number	Name	Length	Sequence
1	A_(765-816)	52	GAAAGCGUGGGGAGCAAACCGGAUUAGAUACCCGGGUAGUCCACGCCCUAAA
2	E_(68)	46	CCGGAAGGUCAAGGGGAGGGGUGCAAGCCCCGAACCGAAGCCCCGG
3	A_(1241-1296)	56	GCCCACUACAAAGCGAUGCCACCCGGCAACGGGGAGCUAAUCGCAAAAAGGUGGGC
4	A_(820-879)	53	GCGCGCUAGGUCUCUGGGUCUCCUGGGGGCCGAAGCUAACGCGUUAAGCGCGC
5	A_(588-651)	64	GCCUGGGGCGUCCCAUGUGAAAGACCACGGCUCAACCGUGGGGGAGCGUGGGAUACGCUC AGGC
6	A_(995-1045)	55	AUGCUAGGGAACCCGGGUGAAAGCCUGGGGUGCCCCGCGAGGGGAGCCCUAGCAC
7	B_(1052-1107)	56	CCAGGAGGUUGGCUUAGAAGCAGCCAUCCUUUAAAGAGUGCGUAAUAGCUCACUGG
8	B_(589-668)	82	CACGGUCGUGGGCGAGCUUAAGCCGUUGAGGCGGAGGCGUAGGGAAACCGAGUCCGAACA GGGCGUCUAGUCCGCGGCCGUG
9	A_(136-227)	93	CCGGAAGAGGGGGACAACCCGGGGAAACUCGGGCUAAUCCCCCAUGUGGACCCGCCCCUU GGGGUGUGUCCAAAGGGCUUUGCCCGCUUCCGG
10	A_(1113-1187)	74	CCCCGCCGUUAGUUGCCAGCGGUUCGGCCGGGCACUCUAACGGGACUGCCCGCGAAAGCG GGAGGAAGGAGGGG
11	B_(865-911)	46	CACUGAUAGGGCUAGGGGGCCCACCAGCCUACCAAACCCUGUCAAA
12	E_(2676-2731)	57	CGCACCUCUGGUUUCCCAGCUGUCCCUCCAGGGGCAGAAGCUGGGUAGCCAUGUGCG
13	E_(99,100,101)	79	GGACCCGGGAAGACCACCCGGUGGAUGGGCCGGGGGUGUAACGCCGCGAGGCGUUGAGCC GACCGGUCCCAAUCGUCC
14	E_(90,91,92)	76	CGGCUCGUCGCAUCCUGGGGCUGAAGAAGGUCCCAAGGGUUGGGCUGUUCGCCCAUUAAA GCGGCACGCGAGCUGG
15	E_(89)	43	GGCUGAUCUCCCCCGAGCGUCCACAGCGGCGGGGAGGUUUGGC
16	D_(8,9,10)	53	AAUGGGGGAACCCGGCCGGCGGGAACGCCGGUCACCGCGCUUUUGCGCGGGGG
17	A_(1420-1480)	56	CGGGCUCUACCCGAAGUCGCCGGGAGCCUACGGGCAGGCGCCGAGGGUAGGGCCCG
18	A_(240-286)	47	CCCAUCAGCUAGUUGGUGGGGUA AUGGCCCACCAAGGCGACGACGGG
19	A_(442-492)	41	CCCGGGACGAAACCCCCGACG AGGGGACUGACGGUACCGGG
20	E_(65,66)	57	ACUGUUUACCAAAAACACAGCUCUCUGCGAACUCGUAAGAGGAGGUAUAGGGAGCGA
21	E_(86,87)	39	GACUGCGAGGCCUGCAAGCC GAGCAGGGGCGAAAGCCGG

Table S2: The mutations with the largest difference from the wild types of 21 ribosomal RNA fragments using RNAmscTV-Curve, RNAdistance and RNApdist

Index number	Name	Length	Mutation with smallest RNAmscTV-Curve	Mutation with largest RNAdistance	Mutation with largest RNApdist
1	A_(765-816)	52	G21A	G7C	C41G
2	E_(68)	46	G19C	G19A	G19C
3	A_(1241-1296)	56	C6A	C14A	G43C
4	A_(820-879)	53	G35C	C4A	C4A

5	A_(588-651)	64	G29A	G29A	G29A
6	A_(995-1045)	55	C24A	C48A	C48A
7	B_(1052-1107)	56	C28G	C28U	A37G
8	B_(589-668)	82	G31C	C17A	G62C
9	A_(136-227)	93	G76C	G10C	G10C
10	A_(1113-1187)	74	G60U	G60U	G60U
11	B_(865-911)	46	G18U	U42G	C32G
12	E_(2676-2731)	57	C26G	C3A	U54A
13	E_(99,100,101)	79	A63G	G9C	G28C
14	E_(90,91,92)	76	C45A	G10C	G43U
15	E_(89)	43	G17U	A23C	G32U
16	D_(8,9,10)	53	C12G	G31C	G6C
17	A_(1420-1480)	56	C21G	C27G	C27G
18	A_(240-286)	47	G16A	G16A	G16A
19	A_(442-492)	41	A36G	C15A	C38A
20	E_(65,66)	57	A53U	G52U	C21A
21	E_(86,87)	39	G10C	U4C	G10A
